# PsycAssist: A Web-Based Artificial Intelligence System Designed for Adaptive Neuropsychological Assessment and Training

**DOI:** 10.3390/brainsci14020122

**Published:** 2024-01-24

**Authors:** Debora de Chiusole, Matilde Spinoso, Pasquale Anselmi, Alice Bacherini, Giulia Balboni, Noemi Mazzoni, Andrea Brancaccio, Ottavia M. Epifania, Matteo Orsoni, Sara Giovagnoli, Sara Garofalo, Mariagrazia Benassi, Egidio Robusto, Luca Stefanutti, Irene Pierluigi

**Affiliations:** 1 Department of Philosophy, Sociology, Education and Applied Psychology, University of Padua, Via Venezia 14, 35131 Padova, Italypasquale.anselmi@unipd.it (P.A.); andrea.brancaccio@unipd.it (A.B.); ottavia.epifania@unipd.it (O.M.E.); egidio.robusto@unipd.it (E.R.); luca.stefanutti@unipd.it (L.S.); 2 Department of Psychology “Renzo Canestrari”, University of Bologna, Piazza Aldo Moro 90, 47521 Cesena, Italy; matilde.spinoso2@unibo.it (M.S.); noemi.mazzoni5@unibo.it (N.M.); matteo.orsoni2@unibo.it (M.O.); sara.giovagnoli@unibo.it (S.G.); sara.garofalo@unibo.it (S.G.); mariagrazia.benassi@unibo.it (M.B.); 3 Department of Philosophy, Social Sciences and Education, University of Perugia, Piazza G. Ermini 1, 06123 Perugia, Italy; giulia.balboni@unipg.it (G.B.); irene.pierluigi@unipg.it (I.P.)

**Keywords:** adaptive assessment, PsycAssist, planning skills, fluid intelligence, knowledge space theory

## Abstract

Assessing executive functions in individuals with disorders or clinical conditions can be challenging, as they may lack the abilities needed for conventional test formats. The use of more personalized test versions, such as adaptive assessments, might be helpful in evaluating individuals with specific needs. This paper introduces PsycAssist, a web-based artificial intelligence system designed for neuropsychological adaptive assessment and training. PsycAssist is a highly flexible and scalable system based on procedural knowledge space theory and may be used potentially with many types of tests. We present the architecture and adaptive assessment engine of PsycAssist and the two currently available tests: Adap-ToL, an adaptive version of the Tower of London-like test to assess planning skills, and MatriKS, a Raven-like test to evaluate fluid intelligence. Finally, we describe the results of an investigation of the usability of Adap-ToL and MatriKS: the evaluators perceived these tools as appropriate and well-suited for their intended purposes, and the test-takers perceived the assessment as a positive experience. To sum up, PsycAssist represents an innovative and promising tool to tailor evaluation and training to the specific characteristics of the individual, useful for clinical practice.

## 1. Introduction

The prototype of a web-based artificial intelligence (AI) system designed for neuropsychological adaptive assessment and training, named PsycAssist (psychological assistant), is presented. The system was designed to overcome some limitations that traditional paper-and-pencil tests and some existing computerized tests have. In fact, the system introduces several noteworthy features compared to existing ones: (a) it presents potential applicability in clinical settings, offering both quantitative and qualitative automatic feedback; (b) it utilizes adaptive assessment algorithms that mitigate the learning effect, address monotony for skilled examinees, and minimize frustration for examinees within clinical populations; (c) it incorporates rigorous and innovative methodological approaches; and (d) its scalability allows for potential enrichment by incorporating additional tests, enhancing its versatility and the scope of the assessments. This is in line with the attention that neuropsychology researchers have recently given to the need for integration between modern psychometric theories and technological advances in neuropsychological assessment (see, e.g., [1,2,3,4]).

PsycAssist was developed and applied within an Italian project funded by the Ministry of Research and University. This project aimed at the development of computerized tools for the adaptive and personalized assessment of executive functions (EFs) and fluid intelligence (FI).

“Executive functions” is an umbrella term that identifies multiple inter-related neurocognitive processes mainly controlled by the prefrontal cortex, such as cognitive flexibility (or set shifting), working memory, planning, problem-solving, inhibition, and interference control [5]. EFs are regarded as high-level mental abilities that regulate lower-level processes [6,7], enabling individuals to plan and organize thoughts in a goal-directed way, to understand complex or abstract concepts, to evaluate and make decisions, and to repress inappropriate behavior [6,7,8]. For these reasons, EFs contribute to an individual’s adaptation to the environment, everyday living, and academic and occupational successes [8]. FI represents the ability to think logically, process new information, learn, and solve problems in novel situations [9,10,11], other than perceiving relations among elements [12]. Deficiencies in EFs and FI occur frequently in neurodevelopmental disorders (e.g., attention-deficit/hyperactivity disorder, autism, learning disabilities), after brain damage, in psychiatric disorders, and in relation to aging (e.g., [6,13,14]). For these populations, the accurate assessment of FI and EFs is extremely relevant since it provides valuable information to plan personalized interventions. However, assessment among these populations might be difficult due to the combination of the length and the need for extended administration time of traditional tests, along with the low attention capabilities of people with the aforementioned conditions. This might lead to assessments that are poorly reliable, marginally accurate, and inefficient. Therefore, the availability of tools that allow for personalized and adaptive assessments would be crucial in order to plan early and tailor-made rehabilitation interventions.

Several tests and questionnaires already exist for the assessment of both EFs and FI. Some examples of the assessment of EFs are the Tower of London test [15], attentive matrices [16], and the Wisconsin card-sorting test [17]. FI assessment examples include Raven’s progressive matrices [18,19]. For some of them, a computerized version exists that allows obtaining immediate feedback on the results. Still, it does not overcome an important inefficiency issue, which is that individuals should answer all the test items regardless of their characteristics. These tests have followed a rigorous validation process with statistical techniques belonging to the classical test theory (see, e.g., [20]) or item response theory [21]. In both cases, limited efforts were made to create adaptive versions of the tests. Ref. [22] introduced an adaptive battery assessing several cognitive functions, but it did not include a measure for planning ability. In contrast, ref. [21] developed the Tower of London Adaptive Test (ToLA) to assess the abilities of individuals with neurodevelopmental disorders efficiently and accurately. However, this version has not undergone clinical studies despite its design. For a complete and in-depth description of these studies, see Section 2.1.

An alternative and more recent methodological approach for building assessment tools is knowledge space theory (KST, see, e.g., [23]). It is a mathematical theory for the efficient assessment of individual knowledge aimed at personalized learning. One of the most important fields of application of KST is the adaptive assessment of knowledge. Adaptive assessment tests allow the evaluation process to be personalized on the basis of the responses an examinee gave to previous questions. The advantages that come from adaptive assessments are many and concern both the examinees and the examiner. Indeed, the number of questions asked decreases, as well as the effort required from each examinee. The effect is that the “quality” of each answer increases, allowing the examiner to collect more reliable data, and, thus, leading to more accurate feedback. Moreover, most EFs and FI standard tests have a so-called “learning effect” problem [24]. This describes a situation in which an individual learns strategies for solving test items during the filling of the test. The consequence is that the assessment is contaminated by the learning skills of the individual other than the variable that the particular test measures. This unwanted effect can be reduced a lot with adaptive administration of the test since a minimum number of questions is administered to individuals. Furthermore, a KST-based assessment generates an output that encompasses both quantitative and qualitative insights. Quantitative feedback facilitates a comparison between the examinee’s performance and their reference population. Meanwhile, qualitative feedback is valuable for pinpointing the strengths and weaknesses of the examinee, aiding in the identification of areas that warrant further exploration. This dual nature of output enhances the depth and comprehensiveness of the assessment process.

Almost all the applications of KST were carried out in the area of knowledge assessment. Nevertheless, recent extensions of the theory have shown that it can be successfully applied in fields like psychological assessment (see, e.g., [25] and the assessment of human problem-solving and planning [26]. This last extension is called procedural KST (PKST; [26]) and provides deterministic and probabilistic models for partially ordering individuals based on their performances in problem-solving tasks. The deterministic models account for both the accuracy of the responses and the sequence of actions made by the problem solver. Probabilistic models consist of latent Markov models that are used as the basis of adaptive assessment algorithms.

PsycAssist is an innovative web-based system featuring a highly flexible and scalable adaptive assessment engine based on KST and PKST algorithms. Its adaptability enables the efficient handling of increasing amounts of data, users, and transactions without compromising performance. The system’s scalability allows it to dynamically adapt and expand in response to growing demands and workloads. Section 3 explains how PsycAssist serves as a comprehensive solution for a diverse range of psychological and neuropsychological assessments, hosting and managing various test types to meet a wide spectrum of assessment needs.

The paper is organized as follows. The state of the art of a specific EF (i.e., planning) and of FI assessment is briefly described in Section 2.1, and those of knowledge space theory and PKST are provided in Section 2.2. Section 3 introduces the architecture of PsycAssist, with particular attention placed on its adaptive assessment algorithms. The two web apps available in the system, named “Adap-Tol” and “MatriKS”, are described in Section 4. The former is an adaptive version of the Tower of London-like test for the assessment of planning skills. The latter is an adaptive version of a Raven-like test for the assessment of FI. In Section 5, results on the usability of Adap-ToL and MatriKS are discussed, from the point of view of both the respondent and the evaluator. Section 6 concludes the argumentation.

## 2. Backgrounds

### 2.1. State of the Art on the Assessment of Planning Skills and Fluid Intelligence

EFs are high-level mental abilities that regulate lower-level processes [6,7]. They allow individuals to plan and organize thoughts in a goal-directed manner, to understand complex or abstract concepts, to evaluate and make decisions, and to suppress inappropriate behaviors [6,7,8]. For these reasons, EFs contribute to individual adaptations to the environment, daily life, and academic and professional successes [8].

Among EFs, planning plays a central role. It is regarded as the ability to identify and organize the sequence of steps required to achieve a goal, typically without a predetermined path [27,28]. Traditional planning tasks, such as tower tests, like the Tower of London (ToL; [15]) and Tower of Hanoi [29], are commonly used in neuropsychological assessments. These tasks involve moving objects from one position to another with specific rules and constraints [30]. Therefore, they require individuals to visualize the necessary course of action before manipulating the materials [31]. Successful completion of these tasks involves the ability to consider the overall situation, define sub-goals, and generate a sequence of moves to achieve them.

Tower tasks have been widely used in research on the planning skills of individuals with typical development [27,32] or clinical conditions, such as neurodevelopmental disorders, focal brain lesions, frontal lobe dementia, Parkinson’s and Huntington’s diseases, and psychiatric disorders [6,13,14,33,34]. Among tower tests, ToL is likely the most used with individuals of different chronological ages and conditions.

Despite the popularity of the ToL task in cognitive and clinical studies of EFs and some attempts at standardization (see, e.g., [24,35,36,37]), the literature shows a notable lack of uniformity in the procedure across studies [38]. Several variants of the ToL have been introduced and used to examine planning abilities in different populations [39,40]. Although some of them are close to the original version, others have implemented major changes concerning, for example, the administration procedure, outcome measures, the number of items, and the material itself, so that the comparison of results across studies is challenging [35,36,38,41].

A growing interest has been placed on the computerized versions of these tests. One widely used computerized version of the ToL is the Stockings of Cambridge (SOC) task, a test of the Cambridge neuropsychological test automated battery (CANTAB; [42]). Despite the advantages of computerized ToL tasks (e.g., ease of presentation, ease of use, and accurate data acquisition), they involve critical differences in the task itself, both physically and conceptually. Moreover, computerized versions proposed so far do not solve the “inefficiency issue” that can significantly affect specific clinical populations. This issue concerns the fact that individuals are asked to answer all the test questions. Conversely, adaptive assessment allows for the personalization of administration by using stimuli that dynamically adapt to an individual’s appropriate challenge level. They offer the potential to shorten test times, improve test accuracy, and reduce the effect of irrelevant variance caused by the frustration of having to answer items that are too difficult [43].

Only a few attempts have been made to develop adaptive tools for the assessment of EFs, particularly planning tasks. For example, in a recent study, a novel adaptive battery for the assessment of EFs, composed of eight tasks, was tested with individuals in middle childhood to measure working memory, context monitoring, and interference resolution, but not planning ability [22]. On the contrary, [21] developed the Tower of London Adaptive Test (ToLA) to efficiently and accurately assess planning in individuals with neurodevelopmental disorders. However, the stimuli presented are apparently different compared to Shallice’s one. To develop the test, the authors first created a precisely calibrated item bank using item response theory and then set a suitable algorithm capable of estimating the participant’s skill level during the test. Unfortunately, the version was only designed but was not subjected to clinical studies.

Concerning FI, its assessment generally occurs through reasoning ability tests, which are considered more culture-fair and less affected by differences in learning experiences, test familiarity, or sociocultural status [44,45]. One of the most well-known tests of this type is the Raven’s Matrices test [18]. This test requires the individual to identify the piece that best completes a visual-spatial matrix from a series of given options. Currently, the Raven’s Matrices test is available in three forms, each composed of stimuli that differ in number and type, and presented in a booklet format. Colored progressive matrices (CPM; [19]) consist of 36 colored items and are intended for kindergarten to middle school-aged children and the elderly; standard progressive matrices (SPM; [18]) are composed of 60 black and white items, designed for individuals from 6 years old and above. Advanced progressive matrices (APM; [19]) are composed of 48 more complex items, allowing for the differentiation of individuals with high cognitive abilities, and are intended for adolescents and adults. Within each form, stimuli are placed in order of increasing difficulty and are organized into different series, assessing specific competencies. For instance, CPM are organized into three series, which evaluate the ability to identify similarities (based on shape, dimension, direction, quantity, orientation, figure/background, and density criteria), the ability to detect symmetry, and conceptual thinking skills (i.e., detection of abstract relations according to ‘operant-deductive’ logic and their retention in working memory), respectively [46].

Efforts to limit administration times have led to the testing of reduced versions with adults [47,48,49,50,51,52], high school and university students [53], and individuals of developmental age [54,55], as well as versions with a fixed administration time [56].

Other attempts have been made to automate the administration of Raven’s Matrices, mainly to standardize administration and scoring procedures. The initial efforts involved automating the SPM using slides and a projector, where the exact same stimuli of the original SPM were used. The selected response to each item was indicated by pressing a button. The time and accuracy of each response were registered [57,58,59]. Later on, SPM [60,61,62] and CPM [63] were administered using computers to school-aged children, high school students, psychiatric patients, federal employees, and undergraduate students. These automated versions differed only in their presentation format from the original ones, hence providing no advantages regarding the test length and the duration of attention focus required to the test-takers.

Attempts to create computerized adaptive versions of Raven’s Matrices date back to the 1980s and 1990s [59,64], and continue today, since computerized adaptive testing provides more accurate FI estimations using a reduced number of administered items (see, e.g., [65]). For instance [64], using Rasch models, created a unique test with SPM and APM items for individuals aged 10 to 16. This version consisted of five practice items from SPM followed by the first test item that was relatively easy for that person’s chronological age. If the individual responded incorrectly to this first item, then the second one—in order of difficulty—was presented. Conversely, the algorithm skipped to the third item, and so on, ending the test when the person answered five items incorrectly in a row.

Using item response theory, the Hansen Research Services Matrix Adaptive Test (HRS-MAT) was developed specifically for individuals with autism spectrum disorder, but is also usable with the general population [66]. It measures nonverbal intelligence using tasks similar to Raven’s Matrices. Similar to the tool developed by [64], the instrument’s adaptive algorithm selects items to administer that are appropriate for the participant’s age (children or adults) and ability level, taking no more than 15 min to complete. However, this HRS-MAT is only available for research purposes and it is not used in clinical settings.

To conclude, given the importance of planning skills and FI and the evaluation of their manifestations in both clinical and non-clinical populations, having precise and efficient tools for their assessment is highly relevant. Furthermore, a helpful resource would be a tool with clear standard administration and scoring procedures that is applicable to different types of populations and capable of adapting to the characteristics of the individuals.

### 2.2. Knowledge Space Theory, Procedural Knowledge Space Theory, and Adaptive Assessment Algorithms

Knowledge space theory (KST, see, e.g., [23,67,68]) is a mathematical theory developed for the efficient assessment of knowledge and personalized learning. One of the most prominent features of KST is that no attempt is made to compute linearly ordered numerical scores for sorting individuals. Rather, the goal of the assessment is to describe “what an individual masters”, which is their *knowledge state*, and “what they are ready to learn”, which is named the *outer fringe* of their knowledge state. Formally, a knowledge state is the set K⊆Q of items that an individual masters in a particular knowledge domain *Q*, and the outer fringe of a knowledge state *K* is the set of all problems in Q∖K, such that K∪{q} is a knowledge state. From a pedagogical point of view, the outer fringe is the set of all problems that the individual can learn individually, allowing for the increase of their own knowledge state.

The collection of all knowledge states existing in a population of individuals is the so-called *knowledge structure* (Q,K). The knowledge structure reflects the precedence relations (e.g., prerequisites) existing among the problems in *Q*. Thus, K is a subset of the power set $2^{Q}$ (i.e., the collection of all the subsets of *Q*) of problems. Let ≺ be a precedence relation defined on *Q*. A subset of *Q* is a knowledge state whenever, for every pair q,r∈Q of problems, if r≺q and q∈K then r∈K. From a practical point of view, this means that *r* is a prerequisite of *q*. As a consequence, the state containing *r* but not *q* does not exist.

To provide an example, consider the knowledge domain Q={a,b,c,d,e} composed of five problems, and assume a precedence relation ≺ for which a≺b≺d and a≺c≺e. The following knowledge structure contains all those subsets of *Q* that are consistent with the precedence relation ≺:

K={∅,{a},{a,b},{a,c},{a,b,c},{a,b,d},{a,c,e},{a,b,c,d},{a,b,c,e},Q}.

**Figure 1:**
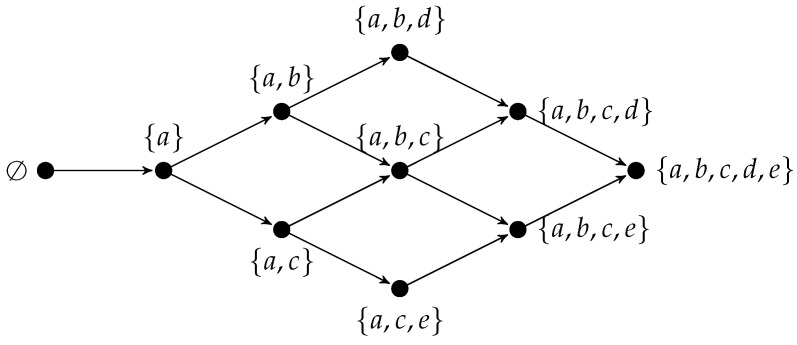
The knowledge structure corresponding to the partial order a≺b≺d, a≺c≺e.

Figure 1 shows the Hasse diagram of K. In the diagram, each node represents a knowledge state, and an arrow from a left node to a right node indicates that the state on the left is a subset of the state to the right.

It can be noted that the empty set (i.e., the situation in which an individual cannot solve any problem) and the full set *Q* (i.e., the situation in which an individual knows everything) are knowledge states. Moreover, among the $2^{5}=32$ different subsets that can be formed with the 5 problems in *Q*, only 10 belong to K. Given that a≺b≺d, all states containing *b* also contain *a*, and all states containing *d* also contain both a,b. A similar reasoning is applied to the other precedence relations.

Assuming that the knowledge state of an individual is K={a,b}, its outer fringe K={c,d} gives information about what the individual is ready to learn (i.e., problems *c* and *d*). Thus, the outer fringe is useful for personalizing learning, and reaching the educational needs of each individual.

Knowledge structures can be empirically validated via probabilistic models. Several probabilistic models are available in the literature [69,70,71,72,73,74,75,76,77] that can be used, depending on the particular application context. Almost all of them are generalizations of the so-called basic local independence model (BLIM; [78]). BLIM is a latent class model, where the latent classes are knowledge states. What is observed is a response pattern (i.e., the subset *R* of items correctly solved) for each individual. The prediction of the knowledge state behind a response pattern is provided by estimating two parameters for each item q∈Q, representing the careless error (i.e., q∈K,q∉R) and the lucky guess (i.e., q∉K,q∈R) probabilities. Throughout the years, BLIM was studied in depth from both theoretical [79,80,81,82] and practical/application [83,84,85] perspectives.

It is worth noting that the original development of KST is useful when dichotomous (correct/wrong) responses are available, and it is specifically meant for the knowledge assessment field of study. In recent years, some extensions of KST were proposed in order to generalize the theory to different fields of applications and different response type formats. For example, the polytomous KST [86,87,88,89,90] allows for nominal, ordered, and partially ordered polytomous response scales.

An extension of KST that is relevant to the project presented in this paper is termed the *procedural knowledge space theory* (PKST; [26]). PKST is useful for the assessment of human problem-solving skills. To this aim, the novelty of the approach consists of modeling the whole solution process made by a problem solver, rather than considering the problem’s accuracy only. PKST is based on both problem space theory [91] and KST. In fact, it provides a formal representation of the notion of *problem space* and makes an algorithm for deriving a knowledge structure starting from a problem space available. The main concepts at the basis of the theory are briefly introduced below.

In PKST, all the problems in *Q* are pairs (s,g), and the objective is to transform an initial configuration *s* into a target configuration *g*. In a problem space, a solution path for a problem (s,g) is a pair sπ where π is the sequence of observable operations required to transform *s* into *g*. It is worth mentioning that a problem (s,g) may have multiple alternative solution paths. Moreover, the solution paths that solve the problems in *Q* are partially ordered. In particular, a solution path sπ is a subpath of another solution path tσ, denoted as sπ⊑tσ if there are two sequences of operations α, and β, such that σ is the sequence απβ, and the sequence α transforms *t* into *s*. The psychological interpretation of the relationship between a solution path and its subpaths is that “if an individual knows a solution path, then they also know all of its subpaths”. This interpretation is named the *problem–subproblem assumption*. The straightforward implication is that an individual who knows how to solve problem (s,t) through the solution path sπ knows how to solve all the problems that are solved by any subpaths of sπ. A whole description of the deterministic foundation of PKST can be found in [26] and in [92].

Just like KST, the deterministic models of PKST need to be empirically validated. The latent class Markov solution process model (MSPM; [92]) can be used for this purpose. The MSPM is useful for making predictions on both the observable solution process and the unobservable knowledge state (the latent class) on which the solution process is built. Under the MSPM, the probability of moving from one configuration of the problem to another one might take one of two alternative forms. One of them is conditional to the belonging of a problem to the knowledge state (i.e., the problem solver is able to plan at least one of the problem’s solution paths) and the other one is conditional to the ‘non-belonging’ of a problem to the knowledge state (i.e., the problem solver is not able to plan any of the problem’s solution paths).

One of the main fields of application of both KST and PKST is the adaptive assessment of knowledge. In this respect, precedence relations in KST and the problem–sub-problem assumptions in PKST play a central role. In brief, an adaptive assessment consists of administering items selected on previously observed responses. This has the effect of personalizing the assessment and minimizing the number of questions. In the KST framework, an example is as follows: If a≺b and an individual provides a correct answer for problem *b*, then it is plausible to assume (in a situation without noise) that she is able to solve item *a*. In the PKST framework, an example is as follows: if problem (s,g) is solved by the path sπ, and tσ⊑sπ (i.e., tσ is a subpath of sπ), then an individual who is able to solve (s,g) is able to solve all those problems that are solved by tσ. Precedent relations, on the one hand, and problem–subproblem assumptions, on the other hand, allow for the prediction of the answer.

In the KST framework, several types of adaptive algorithms have been proposed (see, e.g., [78,93,94,95,96,97]). The most used one is the continuous Markov procedure proposed by [97]. The basic idea is that a likelihood function over the knowledge structure expresses the plausibility of the states. At each step of the assessment process, the likelihood function is updated according to the observed correct or wrong answer via a Bayesian rule that takes into account the careless error and lucky guess parameters of the items. The assessment terminates when the mass of the likelihood is concentrated on a single state, which is regarded as the uncovered state of the individual.

A similar algorithm was proposed by [98] in the PKST framework. The main difference between the KST-based and the PKST-based algorithms is that the latter updates the likelihood on the basis of the whole observed solution process instead of using only the correct/wrong answer. In [98], it was shown that the PKST-based algorithm outperforms the KST-based algorithm in terms of efficiency and accuracy since the former one employs more information from the observed data than the latter one.

## 3. The Architecture of PsycAssist

In this section, a description of the information technology architecture of the system is given. PsycAssist is a highly flexible and scalable web-based system whose *adaptive assessment engine* is based on KST and PKST algorithms. The flexibility and scalability of the system allow it to handle a growing amount of data, users, or transactions without compromising performance. Mostly, the scalability allows the system to adapt and expand as demand and workload increase. Indeed, as explained in the following section, PsycAssist can host and manage many different types of tests for psychological and neuropsychological assessments, making it a comprehensive solution for a wide range of assessment needs. Moreover, the web-based design of PsycAssist facilitates global accessibility for evaluators, allowing them to utilize the system from any location worldwide and on any device, simply connecting to the web page at https://psycassist.fisppa.unipd.it/research_project/). The only requirement is the availability of an internet connection.

The system is designed to cater to a diverse range of professionals who are licensed or authorized to administer psychological tests, including psychologists and neuropsychologists. Users have their own credentials to access the system. Once logged, a user can add a list of test-takers who can be assessed with all the tests available on the system. The system collects and organizes the results of any assessment made by any test-takers. In fact, at the end of any assessment, an automatically generated report describes in detail the results of the assessment by using several types of information, both quantitative and qualitative, which might be of support for clinicians.

It is worth noting that the system is inherently an explainable AI system in two different aspects. In the former, it is a rule-based system in the user interaction since the rules are conditional statements that establish how the system should behave or make decisions in various situations, depending on the answers provided by the user. Section 3.1 describes the rule-based algorithm at the core of the system. The other aspect that makes PsycAssist an AI-based system concerns the so-called knowledge structures. Each test implemented in the system is referred to a specific knowledge structure that represents the organization and the connections of a particular knowledge (or cognitive) domain. These knowledge structures are sometimes constructed by using machine learning methods, such as advanced clustering algorithms (see, e.g., [93,99]).

The subsequent sections offer detailed descriptions of both the assessment engine and the assessment module. Additionally, comprehensive information regarding the compatible types of devices for system usage and the security measures implemented within the system are provided.

### 3.1. The System Engine: A PKST-Based Adaptive Algorithm

**Figure 2:**
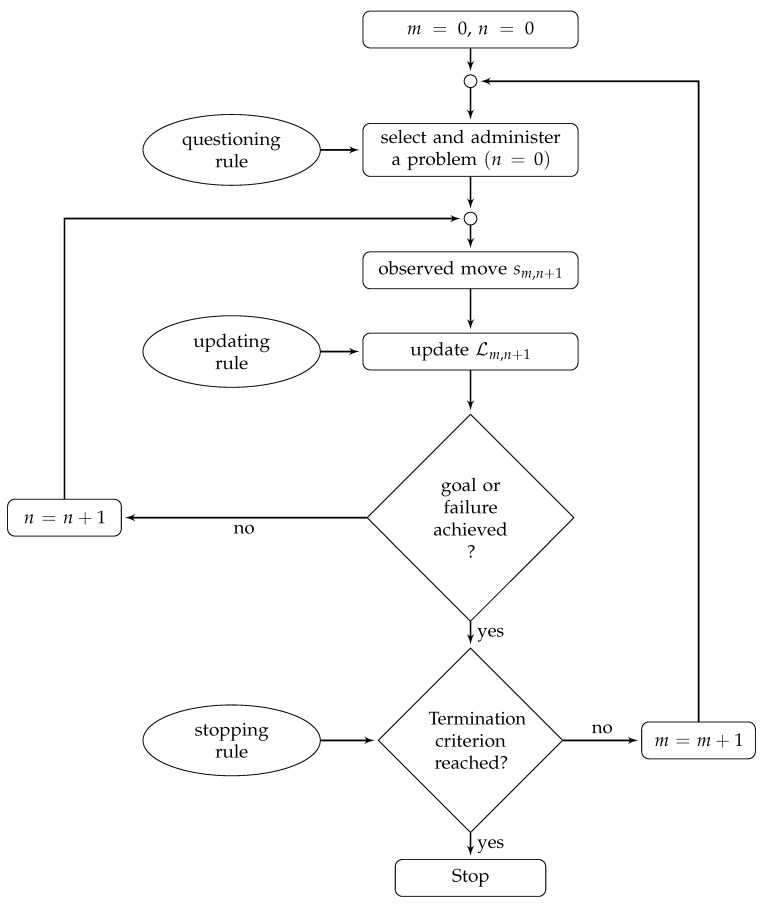
Algorithm of the adaptive probabilistic procedure implemented in PsycAssist. See text for more details.

The assessment engine of PsycAssist is a generalized version of the PKST-based adaptive algorithm proposed by [98]. Figure 2 shows the flowchart of the algorithm.

The algorithm consists of two nested loops. The outer loop handles the administration of a new problem, whereas the inner one handles and records the steps of the solution for the problem. It is worth noting that, in situations in which a single-step answer is required for the problem, the algorithm does not enter the inner loop. In this last case, the PKST-based algorithm is equal to the KST-based one.

The assessment begins with the initial values m=0 and n=0 representing the iterations throughout the outer and inner loops, respectively. At the same starting step, the likelihood $L_{0,0}$ is set to be a uniform distribution among the knowledge states. Starting from $L_{0,0}$, the assessment is carried out iteratively. At each iteration of the external loop, a questioning rule, an updating rule, and a stopping rule are applied.

At each step, *m*, the most informative problem, q∈Q, is selected according to a *questioning rule* named *half-split*. Let $K_{q}$ be the collection of all states containing problem *q*; the problem selected by the half-split is the one that minimizes the difference

$$|L_{m,n}\left(K_{q}\right)-\frac{1}{2}|.$$

If there are several problems that minimize that quantity, then one of the problems is selected at random. In other words, the half-split rule selects the problem for which the two probabilities of the individual responding correctly or incorrectly are as similar as possible. From a psychological point of view, this amounts to selecting a problem that is neither too difficult for the individual (and, thus, demotivating) nor too easy (and, thus, boring).

The participant is presented with the initial configuration of the problem $s_{m,n}$, selected by the questioning rule, and the likelihood $L_{m,n+1}$ is updated on the basis of the participant’s move from the configuration $s_{m,n}$ of the problem to the configuration $s_{m,n+1}$. The *updating rule* computes $L_{m,n+1}$ from the $L_{m,n}$ by

(1) $$L_{m,n+1}\left(K\right)=\frac{P\left(s_{m,n+1}|s_{m,n},q,K\right)L_{m,n}\left(K\right)}{\sum_{K^{\prime }\in K}P\left(s_{m,n+1}|s_{m,n}q,K\right)L_{m,n}\left(K^{\prime }\right)},$$

where $P(s_{m,n+1}|s_{m,n},q,K)$ is the conditional probability of moving from $s_{m,n}$ to $s_{m,n+1}$, given the knowledge state K∈K and problem q∈Q. Practically, the Bayesian updating rule updates the likelihood of all knowledge states in the knowledge structure as follows. If the individual provides a correct answer to the problem, the likelihood of all states containing it is increased, and the likelihood of all states not containing it is decreased. If the individual fails the problem, the likelihood of all states containing it is decreased and the likelihood of all states not containing it is increased.

The procedure continues by selecting and administering problems and by updating the likelihood of the knowledge states until the likelihood of one of them reaches a pre-specified termination criterion. In particular, as soon as the likelihood $L_{m,n+1}\left(K\right)$ of any knowledge state K∈K is greater than a termination criterion p∈(0.5,1], the assessment terminates. Otherwise, an additional problem is administered. Upon termination, the knowledge state whose likelihood is maximum provides an estimation of the knowledge state of the individual.

It is worth noting that [92,98] presented three possible explicit forms for the conditional probability in Equation (1), which are based on three different assumptions, describing different solution behaviors.

What the three assumptions have in common is that the conditional probability $P(s_{m,n+1}|s_{m,n},q,K)$ has two interpretations, depending on the belonging (or not) of problem *q* to the knowledge state *K*. Suppose that $s_{m,n+1}$ is a problem solution, *q*. The probability of making a move from $s_{m,n}$ to $s_{m,n+1}$, given that the individual masters the item (i.e., q∈K) is interpreted as a non-careless error. Conversely, the probability of making a move from $s_{m,n}$ to $s_{m,n+1}$, given that the individual does not master the item (i.e., q∉K), is interpreted as a lucky guess. In this respect, these two interpretations are the same as the careless error and lucky guess parameters of the BLIM.

Instead, the three assumptions differ in the type of planning that is considered. The first assumption, named the *pre-planning assumption*, describes a situation in which a participant meticulously plans out the entire solution process for the problem and applies it at every step. The second assumption, named the *interim-planning assumption*, describes a situation in which several planning instances may take place during the solution process. Finally, the third assumption, named the *mixed-planning assumption*, describes the situation in which both pre-planning and interim-planning might occur. The formal characterization of such assumptions can be found in [98].

The algorithm at the core of PsycAssist is versatile, allowing for personalizations based on the specific aim and task of the test. The two tests currently available on the system were implemented by applying the pre-planning assumption.

### 3.2. The Assessment Module

Regardless of the particular test to be administered, the administration procedure consists of the following four phases: (1) instruction; (2) practice; (3) testing; and (4) conclusion.

The *instruction phase* consists of a short video tutorial to be watched by the test-taker. The tutorial includes all the information useful to complete the task characterizing a test. Specifically, a human-like avatar reads the text on the screen, describing the task, the type of responses the test-taker must provide, and the rules to be respected for accomplishing the task. Concurrently, the video demonstrates the correct execution of the task while the instructions are narrated. To ensure universal accessibility, the video tutorial was crafted by incorporating insights from the literature, drawing from various sources, such as (a) previously developed computerized tests [100,101,102], (b) self-reported measures tailored to individuals with intellectual and developmental disabilities [103,104], and (c) established guidelines in the field [105], including the widely recognized Universal Design for Learning (see, e.g., https://udlguidelines.cast.org/). Key features adopted for enhanced accessibility include the use of a sans-serif font, appropriate font sizes, high-colored contrast between letters and background, spacious layouts without narrow spacing, the absence of hyphenations, the incorporation of visual markers to emphasize essential words (e.g., bold, different colors) or images (e.g., circles, arrows), and the integration of animations without flashes, sparkles, or flickers. Furthermore, the video allows for personalized volume adjustments to cater to individual preferences.

After instructions, a *practice phase* follows. Regardless of the specific test, the trial block comprises a limited number (three or four) of basic test items. The responses to these items do not contribute to the final individual’s assessment. However, they play a crucial role in assessing whether the test-taker masters the essential skills to complete the test. Specifically, if more than 75% of the observed responses are incorrect during the practice phase, the evaluator may opt to forego administering the test.

In the *assessment phase*, individual test items are presented sequentially, one at a time. The modality of responding to these items is contingent on the specific nature of the test. For instance, responses may involve selecting an option or engaging with objects by moving them from one position to another. If a test-taker is unable to provide an answer for a particular item, the evaluator can decide to skip it by pressing the designated button. Throughout the assessment phase, several variables are recorded in a database. The specific variables to be recorded are contingent on the characteristics of the particular test. However, common variables include the order of items, the responses of the test-takers, and the time spent on each item. In terms of sociodemographic information, the system can capture gender, age, nationality, and years of schooling for each test-taker. Notably, the system does not allow the registration of other personal data. A concluding message appears on the screen once the test is completed.

The *conclusion phase* pertains to the evaluator and consists of consulting a report that is automatically generated by the system. In fact, at the end of each administration, the system automatically generates a comprehensive report, offering valuable insights into the performance of the test-taker. The content of the report is contingent on the particulars of the administered test. Regardless of the test, the report furnishes tables and figures illustrating the frequency and percentage of correct, incorrect, and unanswered responses. Furthermore, the report includes more specific descriptions of the results, organized into distinct sections. These sections provide a nuanced understanding of the test-taker’s performance, enhancing the clinicians’ insights into various facets of the assessment.

### 3.3. Device Typologies

As highlighted in the literature, several advantages are linked to computerized tests. These advantages concern (a) the ease of administration (less training, reduced time, accuracy in recording responses) and scoring (no effects of the evaluator’s bias, automated calculation, and presentation of the findings); (b) the reduction of long-term costs; (c) the possibility of instant feedback on individual performance, which is especially useful in a clinical setting; (d) the ease of importing data into statistical software, which is useful in a research setting; (e) instantaneous recording of the performance, which can also be used to tailor the task difficulty to the individual performance level [106,107]. Furthermore, due to the time-efficient method of administration, computerized tests appear ideal if an individual needs to be tested multiple times, for example, to monitor changes and effects of interventions in clinical populations [107].

Concerning the device to be used for administering a computerized test, tablets are particularly useful. Indeed, they are readily available, relatively cheap, and portable, and, compared to smartphone technology, have an adequate screen size for administering cognitive tools [108]. Touchscreen use requires less eye-motor coordination than indirect input devices (mouse/keyboard), and is, thus, particularly useful for patients with mild motor dysfunctions [109]. Moreover, the touchscreen provides a more engaging experience and seems more intuitive even for those unfamiliar with digital devices, as well as for patients with mild cognitive impairment [109,110].

For all these reasons, the usage of a tablet is suggested to administer tests via PsycAssist. However, a computer can be used as well.

### 3.4. Security of the Platform

The platform is equipped with the most recent “safety parameters” according to the European legislation on privacy (see, e.g., the General Data Protection Regulation, GDPR, at https://gdpr-info.eu/, and the Artificial Intelligence Act at https://artificialintelligenceact.eu/)). The precautions and strategies adopted to protect the personal data being processed include techniques such as disk encryption, encryption of individual files, encryption of web communications via the HTTPS protocol, database, and backup encryption, and data pseudonymization to prevent the association between personal data and sensitive data; moreover, access control is enforced through the use of JSON Web Tokens for authentication and authorization operations.

## 4. Web Apps for the Neuropsychological Assessment

Two web apps are currently available on PsycAssist: Adap-ToL, a test for the assessment of planning skills, and MatriKS, a test for the assessment of FI. The following sections describe these two tests.

### 4.1. Adap-ToL: Assessment of Planning Skills

Adap-ToL is a test designed to assess the planning skills of individuals who are four years old and above (children, adolescents, and adults), with or without clinical conditions. Similar to the Tower of London test [15] and other classic tower tests [15,29,111,112], the task is to move beads from an initial configuration to a goal configuration, using a given maximum number of moves and respecting specific rules.

In developing Adap-ToL, some precautions were adopted to maximize comparability with the traditional ToL test [38]. First, the computerized testing material was created to be as faithful as possible to the physical one, with the same number of pegs and beads to be manipulated and, consequently, the one-to-one ratio of beads to empty spaces. Furthermore, the required task and recorded responses (correctness, planning time, execution time, total time, violations, number of moves) are the same. Regarding the rules, those that can be replicated via a digital device were maintained, e.g., (a) the highest peg can hold three beads; the central peg can hold a maximum of two beads, and the shortest peg can hold only one bead; (b) a bead cannot be moved if it has another bead on its top. At the same time, the tool shows some differences from the traditional ToL, which are described below.

Unlike the traditional ToL, in which the initial state is the same for all the items, but the goal state changes, in Adap-ToL, the goal state is fixed for all the problems, while the initial state changes. The reason why this modification was introduced is strictly connected with the probabilistic model MSPM on which the PKST-based assessment procedure is based. In fact, the conditional probabilities used in the adaptive assessment procedure (see Section 3.1) require that the two problems share the same goal.

Moreover, in the original ToL test [15] an indirect measure of the difficulty of a problem is obtained as the minimum number of moves necessary to solve it. However, recent studies [38,41,113] found that other factors affect the difficulty of a problem. Some of them are the number of alternative solutions for the problem, the initial configuration of the beads on the pegs (named “start hierarchy”), and the final configuration (named “goal hierarchy”). PKST goes beyond the notion of the minimum number of moves, considering partial orders of the item’s difficulty instead of a total one.

The partial order of item difficulty built for Adap-ToL takes into account several features of the items. Table 1 lists the 35 items included in Adap-ToL, specifying, for each of them (Columns 1 and 5), the start hierarchy (Columns 2 and 6), the number of moves required for reaching the goal state with the minimum number of moves (Columns 3 and 7), and the number of alternative solutions (Columns 4 and 8). Concerning the start hierarchy, a “1” specifies the so-called ambiguous configuration, a “2” specifies the so-called partial ambiguous configuration, and a “3” specifies the so-called unambiguous configuration. The reader can refer to [114] for a comprehensive description of these configurations.

Adap-ToL can be administered in three different versions. Adap-ToL 4–8 is used with children between the ages of 4 and 8 years old and includes Items 1 to 20 of Table 1. Adap-ToL 9–13 is used with children of age between 9 and 13 years old and includes Items 1 to 27. Finally, Adap-ToL 14+ is used with individuals who are more than 14 years old and includes all 35 items listed in Table 1.

**Figure 3:**
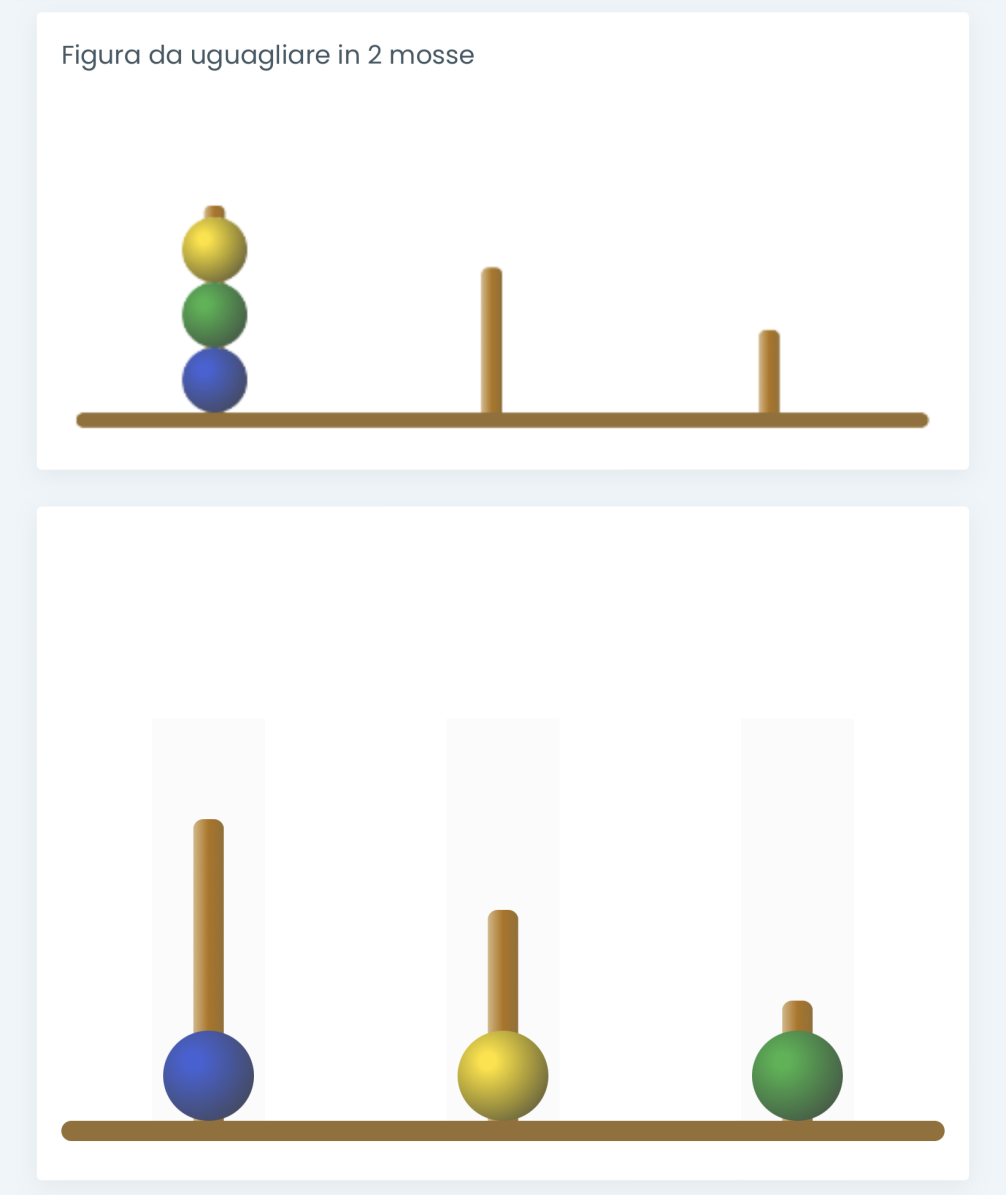
One of the items included in Adap-ToL. The stimulus is composed of two images. The one in the upper part is the goal state and the one in the lower part is the initial configuration. The initial configuration is used by the test-taker for making the moves. The text in Italian “Figura da uguagliare in due mosse” on the left top corner of the figure means “Figure to be matched in two moves”.

In Adap-ToL, the task is to move from an initial state to a goal state using a given maximum number of moves and respecting specific rules. When a tablet is used, orienting the screen vertically is recommended. Two images are presented to the test-taker, one in the upper part (i.e., the goal state) and one in the lower part (i.e., the initial configuration) of the screen. Figure 3 shows the screenshot of one of the Adap-ToL problems.

The initial configuration is used by the test-taker for making the moves. Each of the two images represents a base from which three pegs of different heights rise, into which three colored beads are inserted. Differently from the original ToL, the beads are blue, green, and yellow, with yellow used instead of red to make the instrument accessible to individuals with red–green dyschromatopsia. The number of moves needed to solve the problem successfully appears at the upper left of the screen.

The required movement in Adap-ToL is a double tap on the monitor. With a first touch, the individual selects the bead to move; with a second touch, the individual selects the peg where they want to insert the bead. The problem is correctly solved when the goal state is reproduced using no more than the indicated number of moves.

The system incorporates three types of feedback pop-ups that may appear for the test-taker. Feedback is provided in cases where a rule is violated, when an excessive number of moves are made to complete the task, or when the task is correctly executed. Each feedback is presented within a colored box (red, orange, or green, respectively) to enhance the intuitiveness of the feedback, especially for children who cannot read yet or who may face challenges in reading. In such instances, the evaluator reads the message aloud.

Unlike the traditional ToL, in which three attempts are required for each item, in Adap-ToL, only one attempt is allowed.

Throughout the execution of Adap-ToL, the system records various parameters, including (a) the accuracy of the item, which reflects whether the task is completed correctly or incorrectly; (b) the pre-planning time, which is the duration from the presentation of the stimulus to the initial touch of a bead, indicating the time taken for pre-planning before problem-solving begins; (c) the execution time, which is the duration from the first touch of the bead to the completion of the problem, measuring the time dedicated to executing the task; (d) the total time, which is the cumulative time encompassing both pre-planning and execution phases; (e) the number and type of violations that capture instances where test-takers deviate from established rules, with information specifying the type and frequency of violations; (f) the number and sequence of moves, which detail the quantity and order of movements made by the test-takers during the task. These recorded parameters offer a comprehensive assessment of the test-taker’s performance during the execution of Adap-ToL.

### 4.2. MatriKS: The Assessment of Fluid Intelligence

MatriKS is a test designed to assess FI, specifically targeting reasoning abilities. It was developed considering the principles of Raven’s Matrices. Like Adap-ToL, the idea behind the creation of MatriKS was to have a unique tool usable with different populations: children, adolescents, and adults, with or without clinical conditions.

As in traditional tests that use Raven-like matrices as stimuli, the MatriKS task aims to identify the piece that best completes a visuospatial matrix among the proposed options. Response options in MatriKS can be five or eight, depending on the matrix type. The incorrect options, referred to as “distractors”, were intentionally crafted to replicate the same types of errors observed in traditional Raven’s Matrices [115]. These errors are classified into the following four categories:

1. a *difference* error occurs when the selected distractor exhibits a qualitative difference from the other choices, making it visually distinctive and likely to “pop” among the response options. Examples include a completely blank option or an option featuring extraneous shapes not present elsewhere in the item;
2. a *repetition* error arises when the chosen distractor replicates a matrix tile adjacent to the blank;
3. a *wrong principle* error occurs when the distractor selected is a copy or composition of elements occurring in various tiles of the matrix, combined according to an incorrect rule;
4. an *incomplete correlate* error occurs when the chosen distractor is almost correct but deviates by only one rule.

Categorizing erroneous responses using these classifications facilitates a comparison with the examination of specific error patterns observed in traditional Raven’s Matrices, spanning diverse age groups and clinical conditions [116,117,118]. This approach allows for a meaningful analysis of error trends and patterns, contributing to a broader understanding of cognitive performance across different contexts and populations.

The matrices included in MatriKS, along with their corresponding response lists, were generated using a novel R package designed for the automated creation of Raven-like matrices (MatriKS; [119]). In brief, MatriKS generates stimuli based on the specified parameters, including (a) the dimension of the matrix (e.g., 2×2 or 3×3); (b) the objects to be used (e.g., square, circle, etc.); (c) the rule that guides the manipulation (e.g., change in shape, orientation, size, etc.); (d) the direction of the manipulation (e.g., vertical, horizontal, or diagonal). For a comprehensive understanding of the package and its functionalities, readers can refer to the documentation accompanying the MatriKS package.

Table 2 lists the features of the stimuli included in MatriKS 4–11. Concerning the stimuli included in MatriKS 12+, all of them are in black and white, only three have dimension 2×2 with five response options, whereas all of the others have dimension 3×3 with eight response options. The two versions share 11 stimuli. Stimuli of MatriKS 4–11 require visual–perceptual, elaboration of the general configuration, reasoning, and elementary inference skills. In addition to these skills, stimuli of MatriKS 12+ require advanced skills of reasoning and logic inference.

**Figure 4:**
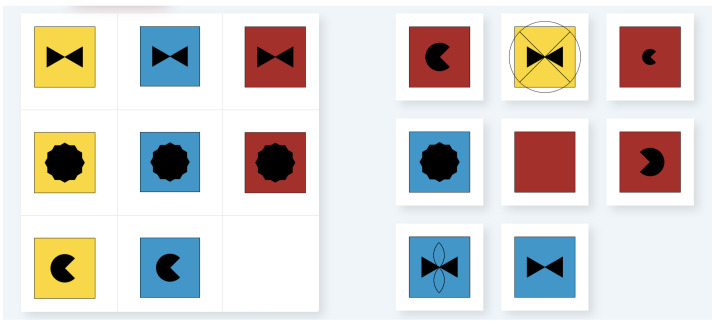
One of the stimuli of MatriKS 4–11, which is a colored 3 × 3 matrix with eight response options. The matrix is presented on the left and the response options are presented on the right. The missing cell is at the low-right corner of the matrix.

The stimuli of MatriKS are composed of two parts: the matrix and the response options. The matrix is presented on the left of the screen and can be of three types: monothematic matrices (named “Mono” in the table), representing a single figure from which a piece is missing; 2 × 2 matrices, series of four cells, one of which is missing; and 3 × 3 matrices, series of nine cells, one of which is missing. The missing cell is at the low-right corner of the matrix. Response options are presented on the right of the screen. To provide the answer, the test-taker selects one of the options. Figure 4 shows one of the stimuli of MatriKS.

When a tablet is used, MatriKS should be administered by orienting the screen horizontally for a better view of the stimuli. The MatriKS trial block consists of two monothematic matrices and two 2×2 matrices. In case of an error in at least one of the two monothematic matrices, the evaluator shows the video tutorial again and presents the same trial items one more time before moving on to the test items.

During the task, the system automatically records correct, incorrect, or non-provided answers. In case of an incorrect answer, the type of response given (i.e., the distractor) is also recorded. Moreover, the time taken to solve each problem, the total time to complete the test, and the average time per item are recorded.

## 5. Usability Study

The perceived “ease of use” and attitude toward new technologies are particularly relevant for the effectiveness and implementation of new digital neuropsychological tests. Understanding and evaluating these aspects is crucial to ensure that technology not only improves assessments but is also perceived as facilitating and effectively used by clinicians and patients. Specifically, acceptability refers to the degree to which a user finds a computer system suitable, agreeable, and satisfactory. It is strictly connected to the concept of usability, which is the extent to which a product can be used by specified users to achieve specific goals with effectiveness, efficiency, and satisfaction in a given context of use [120].

To investigate the usability and acceptability of the new digital assessment web apps Adap-ToL and MatriKS, we developed the Usability and Attitude Scale (UAS), which is a revised version of the system usability scale (SUS; [121]) and the technology acceptance model (TAM; [122]) questionnaires.

The system usability scale (SUS) is a widely used questionnaire for measuring usability [121], operatively defined as the subjective perception of interaction with a system. The SUS is specifically designed to generate a comprehensive single measure of perceived usability. It is known as a “quick” survey scale that allows practitioners to easily and efficiently access the usability of novel technologies. The original SUS includes a mix of positive and negative items and it was structured to control for acquiescence bias and to identify respondents who did not pay attention to the statements. However, several studies highlighted the fact that the inclusion of both positive and negative items can be problematic [123,124]. For instance, it can lower internal reliability [124], distort the factor structure [125,126,127], and increase interpretation problems with cross-cultural use [128]. Moreover, mixed items may lead to difficulty in switching users’ response behaviors and it can increase the cognitive load [129]. This could be particularly true for the clinical and younger population, whose EFs may be impaired or still in development.

Notably, some authors suggested that questionnaires for usability assessment should avoid the inclusion of a mixture of positive and negative items and that researchers who do not specifically need to use the standard SUS should consider using the positive version to reduce the likelihood of response or scoring errors (e.g., [130]). A version of the SUS that includes only positively worded items has been created [131]. Evidence about the positive version of the SUS reliability, validity, and sensitivity has been provided [132]. Moreover, more recently, the equivalence in terms of confidence to measure the subjective usability of two versions of the SUS (mixed and only positive) has been shown, together with the fact that the scores generated from the two SUS versions are comparable (e.g., [129]). The positive SUS appears, hence, as a valuable alternative to the standard SUS and offers the advantages of reduced cognitive load and shifting ability.

### 5.1. The Usability and Attitude Scale (UAS)

UAS is a new questionnaire based on SUS [121,131] and TAM [122,133] that has been structured to achieve brevity and ease of comprehension. It is an 8-item questionnaire, adapted in the Italian language, currently undergoing validation. To date, there are no instruments specifically targeted for developmental age that encompass both the features of usability and acceptability. Given the breadth of our sample (ranging from 4 to 70 years of age), it was deemed beneficial to develop a scale suitable even for younger participants (e.g., brief, employing easy language, incorporating emoticons). Therefore, we decided to design the UAS to be administered to all test-takers, regardless of their chronological age. On the other hand, the original versions of SUS and TAM were administered to the evaluators to assess their perceived usability and acceptability of Adap-ToL and MatriKS, given that they were adults capable of adequately responding to the original versions of the questionnaires.

Concerning the UAS, four items assess the usability based on SUS items and four items assess the acceptability based on TAM items. As mentioned above, UAS was designed primarily for developmental age, especially for children ranging from 4 to 13 years of age. Nevertheless, for the study’s purposes, it was administered to all the study participants (i.e., ranging from 4 to 70 years of age).

The SUS authors advocated for a flexible application of the SUS items according to the context of interest. Thus, only 4 out of 10 SUS items were considered. Specifically, referring to the positive SUS version of [131], only positive assertions were included, as they are more comprehensible for the targeted audience of children (i.e., Items 1, 2, 3, and 7). Moreover, for maximizing the comprehension of the response valence, both for younger and older participants, in the proposed UAS questionnaire a 5-point Likert scale was used as the response scale, where “1” corresponded to “strongly disagree” and “5” corresponded to “strongly agree”. The verbal response options were paired with colored emoticon images. This visual aid was designed to enhance comprehension and engagement in the rating process.

A similar approach was followed in selecting items related to acceptability. The original TAM version consists of 12 items, with 6 evaluating perceived usefulness (PU) and 6 assessing perceived ease of use (PEU). PU refers to the degree to which a person believes that technology will enhance job performance, while PEU is defined as the extent to which a person believes that using technology will be effortless [133]. However, MatriKS and Adap-ToL are digital assessment tools, designed to enhance the job performance of clinicians and experimenters but they do not aim to improve that of the tested participants. For this reason, the UAS questionnaire intended for the test-takers exclusively included items related to PEU, while the usability and acceptability questionnaire filled out by the experimenters included both PU and PEU items. Within the PEU items, the UAS questionnaire selectively incorporated just four items. Specifically, Items 5 and 6 pertained to the perceived ease of use for both oneself and others; Item 7 focused on the perceived pleasantness/fun, while Item 8 addressed intentionality.

Concerning the questionnaire administered to the evaluators, it was the original version of the SUS and TAM questionnaires composed of 22 items overall. The responses to the SUS items were provided by using a 5-point Likert scale, while responses to TAM items were provided by using a 7-point Likert scale.

### 5.2. Participants and Procedure

The UAS intended for the test-takers, was administered to a sample of 1239 participants aged 4 to 70 (47% males and 53% females, age mean =19.43, age standard deviation SD=16.86) of the general population. The descriptive statistics of the test-takers sample are reported in Table A1 of Appendix A.

The study was approved by the ethical committee for the psychological research of the University of Padua. Before participating in the study, participants, or their parents if minors, received detailed information about the study purposes and procedure, providing a signed informed consent, in accordance with the Declaration of Helsinki recommendations.

Data were collected in different regions of the North, Centre, and South of Italy (i.e., Abruzzo, Basilicata, Calabria, Emilia Romagna, Liguria, Lombardia, Marche, Puglia, Toscana, Umbria, Veneto). Participants aged 4 to 18 were recruited in schools (i.e., from kindergarten to high school), while adults were recruited using a snowball sampling procedure. The school principals were contacted beforehand to inform them about the aim of the project and to ask about their willingness to be involved in the project. The informed agreements were given to the parents of the children in each of the interested classrooms. Only the children whose parents signed the informed consent were included in the study. The exclusion criteria were the presence of motor, visual, and auditory impairments that prevented the test-takers from completing the task.

Participants completed different versions of MatriKS and Adap-ToL according to their chronological age, that is 4–11 or 12+ years old for MatriKS, and 4–8, 9–13, or 14+ years old for Adap-ToL. Two UAS were filled out by all participants, one after the completion of MatriKS and another one after the completion of Adap-ToL.

To assess the usability and acceptability of Adap-ToL and MatriKS as perceived by the evaluators, the original version of the SUS and TAM questionnaires were filled out by 12 evaluators overall, aged 23 to 37 (100% females, age mean =26.69, age SD=3.92). Evaluators were asked to complete the SUS and TAM twice, namely the first day and the last day of the data collection (pre-test and post-test hereafter, respectively). Evaluators were interns, doctoral students, and research fellows in the field of psychology. All study participants and evaluators completed the questionnaires using a tablet.

### 5.3. Data Analysis

To determine the UAS score, we adopted two different procedures for usability and acceptability. In usability, we adopted the method described in [130] and the curved grading scale of [134,135], both reported below. Concerning acceptability, we applied the procedure outlined by the original authors of TAM [136] to provide acceptability scores consistent with usability metrics.

The Sauro–Lewis curved grading scale (CGS), as proposed by [134,135], offers a useful framework for interpreting these overall scores. CGS utilizes an 11-grade scale, ranging from F (an SUS total score of less than 51.6) to A+ (a SUS total score of less than 84.1). The average value on this scale is 68, corresponding to a grade of C. Table A2 in the Appendix A shows the numerical score range corresponding to each grade. However, this method does not allow for the establishment of targets for other and more specific experience attributes.

Ref. [130] proposed item-level benchmarks estimated through regression analysis to consider specific items from the SUS without adversely affecting the overall score. The authors estimated regression coefficients for each item in the original SUS version to establish relationships between the items and their corresponding SUS total scores. Thus, in accordance with [130], we employed item-level benchmarks to assess the different weight of usability items within the UAS for both Adap-ToL and MatriKS and for each different version of the tests, by using the following formula:

(2) $$x_{ij}=\frac{\mu_{ij}-\alpha_{i}}{\beta_{i}},$$

where $\mu_{ij}$ is the mean of item *i* for the test *j*, and $\alpha_{i}$ and $\beta_{i}$ are, respectively, the intercept and the regression coefficient estimated for item *i*, according to [130].

The application of this procedure allows for determining the estimated value for each SUS item integrated into the usability and attitude scale (UAS), by utilizing the item-level regression coefficients proposed by [130]. Then, we compared the obtained score with the regression coefficients provided by the authors, in order to identify the most relevant items for evaluating the usability of our novel assessment tool.

To provide acceptability scores consistent with usability metrics, first, the initial 1 to 5 responses were converted into scores ranging from 0 to 4. Then, the average obtained on the four items was multiplied by 100/4, where 4 is the maximum value of the scale 0 to 4. The outcome of this calculation yields an acceptability average score that spans from 0 (suggesting very poor perceived usability) to 100 (indicating excellent perceived usability).

Moreover, the item-level acceptability score was computed. To the best of our knowledge, there are no studies that estimated the item-level benchmark for the TAM, as [130] did for the SUS. However, to be consistent with the usability results provided here, and for descriptive purposes, we calculated the single-item mean values of the acceptability as perceived by the participants of the sample. The scores were averaged separately for Adap-ToL and MatriKS, in every version of the two tests, both for the users and experimenters. Then, to obtain 0–100 scores that were comparable with the usability ones, first, the initial 1 to 5 responses were converted into scores ranging from 0 to 4, then these values were multiplied by 100/4 for the users who completed the UAS and by 100/6 for the examiners who completed the original TAM (4 and 6 were, respectively, the maximum value of the response scale). Finally, we assigned the respective A–F grades [134,135].

### 5.4. Results

#### 5.4.1. Perspective of the Test-Takers

Table 3 displays the results concerning the system’s perceived usability of both Adap-ToL and MatriKS, separately for each version of the instruments. To facilitate comprehension, the results are described referring to the CGS solution proposed by [134,135].

The results suggest that both Adap-ToL and MatriKS are well-received by users, regardless of the test’s version. In particular, the perceived usability of Adap-ToL ranged from “more than excellent” (Grade A: Adap-ToL 4–8 and 9–13 versions) to “excellent” (Grade B+: Adap-ToL 14+ version). Concerning MatriKS, the perceived usability resulted in “excellent” for the version tailored to the younger population (Grade A−: MatriKS 4–11), while the usability score of the other version resulted between “sufficient” and “good” (MatriKS12+: Grade between C− and D).

The results obtained with the item-level benchmark method confirmed different weights of usability items within the UAS for both Adap-ToL and MatriKS. Specifically, in the item scores of all three versions of Adap-ToL, Item 1 (“I would gladly take the test again”, translated to Italian as: “Rifarei volentieri la prova”) obtained an A+ grade score, demonstrating consistent excellent values. In contrast, Item 2 (“The test was very easy”, in Italian: “La prova è stata molto facile”) obtained varying scores, ranging from A− in the 4–8 version to C+ in the 9–13 version, and C in 14+ version. Item 3 (“I think that anyone could take this test”, in Italian: “Penso che tutti possano fare questa prova”) obtained a C+ both in the 4–8 and in 14+ versions and a B grade in the 9–13 version. Finally, Item 4 (“It was easy to understand what to do during this test”, in Italian “E’ stato facile capire cosa fare durante la prova”) received a B+ grade in the 4–8 version and A grade in both 9–13 and 14+ versions.

In the MatriKS item scores, Item 1 achieved an outstanding A+ grade in the 4–11 version and a commendable B grade in the 12+ version. Item 2 received a B grade for the 4–11 version but performed poorly, earning an F, in the 12+ version. Item 3 garnered a B− grade for the 4–11 version and a D grade for the 12+ version. Lastly, Item 4 obtained an A− grade for version 4–11, while registering a score between C− and D for version 12+.

Overall, the item score results for Adap-ToL and MatriKS indicate satisfactory usability of the instruments, especially for the population with lower school grades, as evidenced by the greatest UAS usability scores for the Adap-ToL and MatriKS versions designed for this demographic.

Concerns arise regarding the usability scores for higher school grades, as particularly highlighted by Item 2, which showed low scores in Adap-ToL 14+ and MatriKS 12+. Additionally, a notably poor score is observed in Item 3 of MatriKS 12+. A potential explanation for the low score in Item 2 might be attributed to a misinterpretation of the sentence, which could have directed respondents’ attention more toward the task itself rather than the tools. A similar consideration applies to Item 3, where the complexity of the task might have influenced respondents and guided their responses, diverting their attention away from the instruments themselves.

Generally, the lower scores observed in the higher school-grade versions of both tests may be attributed to the extended duration and increased difficulty of their items when compared to those featured in the versions designed for younger grades.

Table 4 displays the results concerning the perceived system acceptability of both Adap-ToL and MatriKS, separately for each version of the instruments.

In both Adap-ToL and MatriKS, the results obtained with the CGS method confirm positive ratings similar to those found for usability. Moreover, the results obtained with the item-level benchmark method show that in all three versions of Adap-ToL, Item 5 “I think that taking the test on the tablet was easy” (in Italian: “Penso che fare la prova sul tablet sia stato semplice”) consistently demonstrated excellent values, from A to A+ grade for all three versions. In contrast, Item 6 “I think that it would be easy for anyone to take the test on the tablet” (in Italian: “Penso che per tutti sia facile fare la prova con il tablet”) exhibited scores in versions 4–8 corresponding to C grades and version 14+ received a B grades. Item 7 “I felt good taking the test on the tablet” (in Italian: “Sono stato bene a fare la prova sul tablet”) attained an A+ grade for all the tests. Finally, Item 8 “I would like to always use the tablet for this test” (in Italian “Vorrei usare sempre il tablet per fare questa prova”) received an A grade for versions 4–8, a B+ grade for versions 9–13, and A grade for 14+ versions.

In the item scores of both versions of MatriKS, Item 5 achieved an outstanding A+ grade in the 4–11 versions and an A grade in the 12+ versions. Item 6 received a C grade for the 4–11 versions and a C+ grade, in the 12+ versions. Item 7 showed an A+ grade for both the 4–11 and 12+ versions. Lastly, Item 8 obtained an A grade for the 4–11 versions, while registering a score of A− for the 12+ versions.

#### 5.4.2. Perspective of the Evaluator

Table 5 shows the results of the perceived usability scores provided by the evaluators for Adap-ToL and MatriKS.

The results suggest that the evaluators perceived excellent usability of both Adap-ToL and MatriKS. Specifically, upon examining potential differences in U-scores between the pre-test and post-test experiences of the evaluators, a decrease of −0.1% was observed for Adap-ToL and an increase of 2.85% for MatriKS. The slight decrease for Adap-ToL in the post-test was associated with Item 4, which states, “I think I would need the support of someone who already knows how to use it” (translated from Italian: “Penso che avrei bisogno del supporto di una persona che sia già in grado di utilizzarlo”). This item exhibited a reduction of 16.04% in the post-test compared to the pre-test, while item 9: “I felt very confident with the test during use” (translated from Italian: “Ho avuto molta confidenza con il test durante l’uso”), showed the principal improvement, demonstrating an increase of 18.77%. The increase in the post-test scores for MatriKS was primarily influenced by the improvements in Items 3 (8.40 %), 5 (11.72 %), 9 (7.91%), and 10 (12.43), whereas minor reductions were noted in Items 6 (−8.55 %), and 7(−7.44 %).

Table 6 shows the results of the acceptability perceived by the evaluators for both Adap-ToL and MatriKS.

The acceptability perceived by the evaluators was excellent and obtained an A+ grade for both Adap-ToL and MatriKS. A decrease between the pre-test and post-test acceptability scores was observed for both Adap-ToL (−1.32%) and MatriKS (−1.32%). Nevertheless, for both Adap-ToL and MatriKS, a within-subjects ANOVA did not reveal statistically significant differences between the pre-test scores and the post-test scores.

## 6. Discussion

PsycAssist is an innovative and flexible web-based AI system designed for neuropsychological adaptive assessment and training. The adaptive assessment engine of PsycAssist draws on PKST [26], a formal approach that furnishes deterministic and probabilistic models for partially ordering individuals based on their performances in problem-solving tasks. Moreover, the web-based design of PsycAssist allows users (e.g., psychologists and neuropsychologists) to utilize the system from any location worldwide and on any device.

The system’s adaptivity and serviceability are helpful, especially for the psychological assessment of specific clinical populations. Indeed, evaluating individuals with specific disorders or conditions can be challenging, often due to their limitations in answering conventional test formats. Adaptive assessments allow for the personalization of the administration, shortening test times, improving test accuracy, and reducing the effect of frustration [43].

When introducing new digital neuropsychological assessment tools, it is crucial not only to conduct psychometric validation of the assessment tests but also to assess the perceived “ease of use” and “attitudes” toward the software by both the test-takers and the evaluators. These aspects are crucial to ensure that technology not only enhances assessments but is also perceived as a facilitative and effective tool by both evaluators and test-takers.

The paper presented here describes the general functioning of the system and the initial results regarding the usability and acceptability of the two web apps available on the system. The results confirmed the positive reception of these two tests, indicating that evaluators perceived these tools as appropriate and well-suited for their intended purposes, and that test-takers viewed the assessment as a positive experience. Despite the overall positive satisfaction, some results provided valuable insights for enhancing and optimizing the usability and acceptability of Adap-ToL and MatriKS in future applications. Furthermore, with regard to the sample of test-takers, it is important to acknowledge a limitation in the usability study. Most of the participants were recruited from schools (N=902), and only a fourth of the participants were adults (N=337). To enhance the comprehensiveness of our findings and gather more insights into perceived acceptability and usability among adults, future research should aim to include a broader representation of older participants.

Moreover, because the examiners were not healthcare providers, conducting a further study with practicing clinicians would be appropriate to verify their willingness to use these instruments in their daily practice. Furthermore, it must be noted that the versions of MatriKS and Adap-ToL used in this usability study were preliminary versions whose validations are in progress. Despite these limitations, feedback provided by both test-takers and evaluators suggests that the new digital neuropsychological assessment tools are promising and deserve further research.

## 7. Conclusions

The use of the system might offer various clinical advantages that warrant attention. First, its automatic report generation and scoring alleviate the evaluator’s burden, significantly reducing the likelihood of errors. This not only enhances the efficiency of the assessment process but also ensures more reliable outcomes. Secondly, the system serves as a versatile tool that complements traditional tests. It offers a detailed exploration of the areas under investigation, providing a comprehensive understanding of the individual’s cognitive profile. The integration of this system with traditional assessments enhances the richness of the evaluative process. A noteworthy aspect is the motivational impact on examinees. The digital format, as opposed to traditional paper–pencil tests, tends to engage examinees more effectively. This is particularly beneficial for skilled examinees who might find traditional methods monotonous. Additionally, it minimizes frustration among examinees within clinical populations, contributing to a more positive testing experience. Lastly, the personalized and qualitative nature of the feedback generated by the system holds significant potential. This tailored feedback not only aids in understanding an individual’s strengths and weaknesses but also provides valuable insights for training and treatment strategies. The ability to monitor the effectiveness of interventions and make adjustments based on personalized feedback adds a dynamic dimension to the potential clinical applications of the system.

## Figures and Tables

**Table 1:** The 35 problems of Adap-ToL with their characteristics. Columns 1 and 5 display the number of the problem, Columns 2 and 6 display the hierarchy of the initial state (see text for more details), Columns 3 and 7 display the minimum number of moves for solving the problem. Finally, Columns 4 and 8 display the number of alternative solution paths.

Problem	Hierarchy	# Moves	# Paths	Problem	Hierarchy	# Moves	# Paths
1	2	1	1	19	2	5	1
2	2	1	1	20	1	5	2
3	2	2	1	21	3	6	1
4	1	2	1	22	2	6	1
5	1	2	1	23	3	6	2
6	2	3	1	24	2	6	2
7	2	3	1	25	3	6	1
8	2	3	1	26	2	6	1
9	2	3	1	27	2	6	2
10	2	3	1	28	2	7	1
11	2	3	1	29	1	7	3
12	1	4	1	30	1	7	1
13	2	4	1	31	3	7	2
14	3	4	2	32	2	7	2
15	2	4	1	33	2	8	2
16	2	4	1	34	2	8	3
17	2	5	1	35	2	8	3
18	2	5	2				

**Table 2:** The 40 stimuli included in MatriKS 4–11. For each stimulus, information concerning color (columns 2 and 6), dimension (columns 3 and 7), and the number of response options (columns 4 to 8) are provided.

# Matrix	Color	Dim.	# Options	# Matrix	Color	Dim	# Options
1	yes	Mono	5	21	yes	2×2	5
2	yes	Mono	5	22	yes	2×2	5
3	yes	Mono	5	23	no	2×2	5
4	yes	Mono	5	24	no	2×2	5
5	yes	Mono	5	25	no	2×2	5
6	yes	2×2	5	26	yes	3×3	8
7	yes	2×2	5	27	yes	3×3	8
8	yes	2×2	5	28	yes	3×3	8
9	yes	2×2	5	29	yes	3×3	8
10	yes	2×2	5	30	yes	3×3	8
11	yes	2×2	5	31	yes	3×3	8
12	yes	2×2	5	32	yes	3×3	8
13	no	2×2	5	33	no	3×3	8
14	no	2×2	5	34	no	3×3	8
15	no	2×2	5	35	no	3×3	8
16	no	2×2	5	36	no	3×3	8
17	no	2×2	5	37	no	3×3	8
18	no	2×2	5	38	no	3×3	8
19	no	2×2	5	39	no	3×3	8
20	yes	2×2	5	40	no	3×3	8

**Table 3:** Results concerning the users’ usability of Adap-ToL (Rows 2 to 4) and MatriKS (Rows 5 and 6). Column 1 lists the version of the test. Columns 2 to 5 list the item-level usability scores. Columns 6 lists the average usability (U-score) scores.

Version	Item 1	Item 2	Item 3	Item 4	U-Score
Adap-ToL 4–8	97.50	80.75	72.46	78.24	82.24
Adap-ToL 9–13	94.09	72.02	75.71	82.78	81.15
Adap-ToL 14 +	92.27	66.00	71.79	81.13	77.80
MatriKS 4–11	93.41	76.11	72.73	79.69	80.43
MatriKS 12 +	75.76	51.19	58.04	65.56	62.64

**Table 4:** Results concerning the users’ acceptability of Adap-ToL (Rows 2 to 4) and MatriKS (Rows 5 and 6). Column 1 lists the version of the test. Columns 2 to 5 list the acceptability item scores. Column 6 lists the average acceptability (A-score) scores.

Version	Item 5	Item 6	Item 7	Item 8	A-Score
Adap-ToL 4–8	83.70	70.26	88.93	81.92	81.20
Adap-ToL 9–13	85.85	71.81	87.98	77.68	80.83
Adap-ToL 14+	86.86	74.34	86.82	81.87	82.47
MatriKS 4–11	84.65	70.75	86.5	81.05	80.74
MatriKS 12+	83.14	71.62	84.65	79.22	79.66

**Table 5:** Results concerning the evaluators’ usability of Adap-ToL (Columns 2 and 3) and MatriKS (Columns 4 and 5). Columns 2 and 4 refer to the first day of the administration (pre-test), whereas Columns 3 and 5 refer to the last day of the administration (post-test). Rows 2 to 11 display the usability item scores. Row 12 displays the average usability scores (U-score). The scores of the items marked with a star are inverted.

Original Item	Adap-ToL		MatriKS	
	Pre-Test	Post-Test	Pre-Test	Post-Test
1. I think that I would like to use the system frequently	100.00	100.00	98.25	100.00
2*. I found the system unnecessarily complex	92.06	87.95	80.35	83.69
3. I thought the system was easy to use	92.38	93.97	83.61	90.63
4*. I think that I would need the support of a technical person to be able to use the system	92.28	75.61	89.33	86.37
5. I found the various functions in this system were well integrated	97.88	99.41	88.28	100.00
6*. I thought there was too much inconsistency in this system	85.43	83.92	90.16	83.06
7. I would imagine that most people would learn to use this system very quickly	91.76	91.01	89.70	83.49
8*. I found the system very awkward to use	88.33	87.87	88.31	84.93
9. I felt very confident using the system	80.05	94.91	87.56	95.08
10. I needed to learn a lot of things before I could get going with this system	81.80	82.83	79.55	90.84
U-score	90.20	89.75	87.51	90.08

**Table 6:** Results concerning the evaluators’ acceptability of Adap-ToL (Columns 2 and 3) and MatriKS (Columns 4 and 5). Columns 2 and 4 refer to the first day of the administration (pre-test), whereas Columns 3 and 5 refer to the last day of the administration (post-test). Rows 2 to 13 display the acceptability item scores. Row 14 displays the average acceptability scores (A-score). The scores of the items marked with a star are inverted.

Original Item	Adap-ToL		MatriKS	
	Pre-Test	Post-Test	Pre-Test	Post-Test
1. My interaction with the system is clear and understandable	96.43	97.62	92.86	96.43
2. Interacting with the system does not require a lot of my mental effort	67.86	80.95	83.33	84.52
3. I find the system to be easy to use	94.05	97.62	90.48	94.05
4. I find it easy to get the system to do what I want it to do	79.76	91.67	82.14	86.90
5. Computers do not scare me at all	94.05	95.24	90.48	90.48
6*. Working with a computer makes me nervous	90.48	98.81	91.67	97.62
7*. Computers make me feel uncomfortable	95.24	95.24	90.48	96.43
8. I find using the system to be enjoyable	84.52	84.52	84.52	78.57
9*. The actual process of using the system is pleasant	96.43	91.67	90.48	83.33
10. I have fun using the system	95.24	86.90	89.29	80.95
11. Assuming I had access to the system, I intend to use it	97.62	92.86	90.48	89.29
12. I plan to use the system in the next four months	92.86	57.14	94.05	53.57
A-score	90.38	89.19	89.19	86.01

## Data Availability

The data presented in this study are available upon request from the corresponding author. The data are not publicly available due to their preliminary nature.

## Abbreviations

The following abbreviations are used in this manuscript:

EF	executive function
FI	fluid intelligence
ToL	Tower of London
KST	knowledge space theory
PKST	procedural knowledge space theory
CGS	curved grading scale
SUS	system usability scale
UAS	usability and attitude scale
PU	perceived usefulness
PEU	perceived ease of use
TAM	technology acceptance model

## Appendix A

**Table A1:** Descriptive statistics of the test-takers sample.

Age (Years Old)	N	Mean	Standard Deviation
4–6	142	5.07	0.76
7–10	284	8.59	1.12
11–13	282	12.13	0.77
14–19	194	15.21	1.27
20–29	85	24.98	2.61
30–39	79	33.8	2.67
40–59	88	49.41	5.98
60–70	85	63.61	3.04
Total	1239	19.43	16.86

**Table A2:** Sauro–Lewis Curved Grading Scale (CGS) that establishes a correspondence between grade and numerical score range [134,135].

Letter Grade	Numerical Score Range
A+	84.1–100.0
A	80.8–84.0
A−	78.9–80.7
B+	77.2–78.8
B	74.1–77.1
B−	72.6–74.0
C+	71.1–72.5
C	65.0–71.0
C−	62.7–64.9
D	51.7–62.6
F	00.0–51.6
